# Octagonal Defects at Carbon Nanotube Junctions

**DOI:** 10.1155/2013/658292

**Published:** 2013-09-05

**Authors:** W. Jaskólski, M. Pelc, Leonor Chico, A. Ayuela

**Affiliations:** ^1^Institute of Physics, Faculty of Physics, Astronomy and Informatics, Nicolaus Copernicus University, Grudziądzka 5, 87-100 Toruń, Poland; ^2^Instituto de Ciencia de Materiales de Madrid (ICMM), Consejo Superior de Investigaciones Científicas (CSIC), C/Sor Juana Inés de la Cruz 3, 28049 Madrid, Spain; ^3^Centro de Física de Materiales CFM-MPC CSIC-UPV/EHU, Donostia International Physics Center (DIPC), Departamento de Física de Materiales, Facultad de Químicas, UPV-EHU, 20018 San Sebastián, Spain

## Abstract

We investigate knee-shaped junctions of semiconductor zigzag carbon nanotubes. Two dissimilar octagons appear at such junctions; one of them can reconstruct into a pair of pentagons. The junction with two octagons presents two degenerate localized states at Fermi energy (*E*_*F*_). The reconstructed junction has only one state near *E*_*F*_, indicating that these localized states are related to the octagonal defects. The inclusion of Coulomb interaction splits the localized states in the junction with two octagons, yielding an antiferromagnetic system.

## 1. Introduction

Carbon nanotubes can behave as metals or semiconductors depending on their geometry and diameter. In fact, small variations on their geometry can lead to quite different electronic characteristics. The possibility of joining different tubes has been an early theoretical proposal [1–3], later experimentally verified [4]. To achieve such junctions, topological defects have to be introduced in the hexagonal carbon lattice.

Indeed, a large number of works have been devoted to the study of electronic and transport properties of single interfaces [5–7], double junctions [8], quantum dots [9], and carbon nanotube superlattices [10, 11]. Junctions in such systems are usually realized by introducing pentagon/heptagon (5/7) topological defects at interfaces between the nanotubes, although other proposals involving different kinds of defects, such as octagons, have also been made [12]. Interface states appearing at nanotube junctions have been customarily related to these topological defects. However, it has been recently shown that such states do not originate from the 5/7 defects, but they are due to the edge-localized states, which in turn originate from the zigzag edges of the joined tubes [13, 14]. Octagons have been proposed as a means of producing T- or Y-junctions, and they can also appear in the connection between tubes of dissimilar radii [15–17].

Octagonal defects forming linear grain boundaries in graphene and in carbon nanotubes have recently been the focus of attention. Experimental works show that such grain boundaries may act as quasi-one-dimensional metallic wires [18]. These grain boundaries can also contain pentagon pairs, but when they are built of octagons only, they lead to spontaneous magnetization [19]. We have theoretically shown that the flat bands appearing at octagonal defect lines are directly related to graphene edge states [14, 19].

In this paper we investigate the electronic and magnetic properties of octagonal rings when they appear surrounded by hexagons. We choose a junction between two zigzag nanotubes, namely, the (8,0)/(14,0) case. These tubes are joined by two topological defects which are spatially separated: an octagon and a pair of pentagons. If further we break the bond shared by these two pentagons we will get another octagon, which has two nodes with only two neighbors. We show that these octagonal defects lead to state localization at the Fermi energy.

## 2. System and Geometry

**Figure 1:**
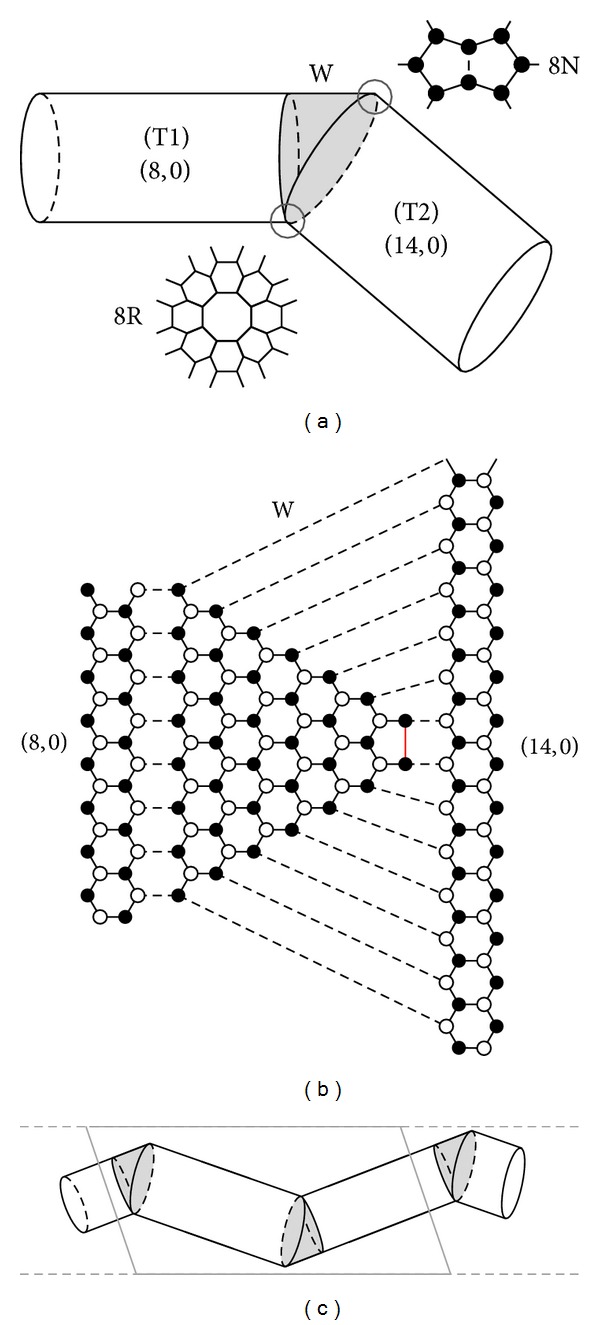
(a) Schematic diagonal junction between zigzag (8,0) and (14,0) nanotubes. The shaded region is the wedge (W) part between the two straight-cut tubes. The circles mark the positions of the octagonal defects 8R and 8N. Their atomic structure is also shown. (b) The junction showing all the edge atoms in the wedge and the unrolled unit cells of the (8,0) and (14,0) tubes. Filled and empty circles mark atoms of different sublattices. Dotted lines indicate the connections between the edge atoms of the nanotubes and the wedge. Short bold, red line in the right apex of the wedge marks the connection which reconstructs the 8N octagon into a pair of two pentagons. (c) Schematic unit cell of the (8,0)/(14,0) superlattice, with the two complementary wedges inside.

It is possible to create junctions between (2*n*, 0) and (4*n* − 2,0) tubes (*n* > 1) by cutting obliquely the (2*n*, 0) tube along a diagonal zigzag direction and joining it to a straight-cut (4*n* − 2,0) nanotube. This produces a knee-shaped junction. We choose *n* = 4, assuring that both tubes are semiconductors. This simplifies the analysis of the zero energy states, which will appear in the gap in these instances. The schematic geometry of the (8,0)/(14,0) case is shown in Figure 1. The oblique connection leads to the appearance of two topological defects: one at the back of the knee and the other at the kneecap. The first is a regular octagon (8R), with all its atoms having coordination number 3; the second is either a pair of pentagons or a nonregular octagon with two dangling bonds, denoted as 8N. This junction can be considered as formed by the connection of three parts, namely, a perpendicularly cut (8,0) tube, a wedge (W) of this (8,0) nanotube, and a straight-cut (14,0) nanotube. The wedge part is shown in Figure 1(a) as a shaded region, along with the detailed atomic geometry of the defects.  Figure 1(b) shows the connection between the two tubes in a planar geometry, that is, their unrolled unit cells and the flattened wedge. The edge atoms are highlighted with filled and empty circles, which represent the two atomic sublattices. Notice that a finite portion of an (8,0) tube diagonally cut at both ends can be joined to two (14,0) tubes. Such double junction has two mirror-symmetric wedge parts, with complementary orientation. The periodical repetition of this double junction yields a superlattice (SL), as shown in Figure 1(c), where the boundaries of a possible unit cell are indicated and the two wedges with mirror symmetry are highlighted. We study both cases, namely, the single junction depicted in Figure 1(a), where the outer tubes are semi-infinite, and superlattices of different sizes.

## 3. Model and Computational Details

We use a one-orbital *π*-electron tight-binding (TB) model. This approach has been extensively employed to calculate the electronic properties of carbon-based systems around the Fermi energy [10, 20]. The hopping parameter is chosen to be *t* = −2.7 eV. We have checked that the changes in *t* induced by the defects amount to a negligible change in the calculated energy spectra, in agreement with previous calculations [19, 20].

In order to see the role of electron-electron interactions in the zero-energy states, we compare the one-electron tight-binding results with those including a Hubbard term. The Hubbard Hamiltonian in a mean-field approximation is given by [21]

(1) H=t∑〈i,j〉,σciσ†cjσ+H.c.+U∑i(ni↑〈ni↓〉+〈ni↑〉ni↓),

where *c*_*iσ*_^†^(*c*_*iσ*_) are the creation (annihilation) operators for electrons with spin *σ* at site *i*; 〈*i*, *j*〉 indicates that the sum takes place within nearest neighbors; *i* is the atom index; and the arrows correspond to the two spin states. The value of the Coulomb repulsion parameter *U* is chosen to be *U* = 3 eV. This choice has been discussed in a number of previous works [19, 22–24]; it is considered to be a reasonable assumption for graphene-based materials. The expectation values of the spin-resolved densities at site *i*, 〈*n*_*i*,*σ*_〉 = 〈*c*_*i*,*σ*_^†^ *c*_*i*,*σ*_〉, depend on the eigensolutions of the Hamiltonian, so the above equation has to be solved iteratively.

For the single junction, we employ a Green's function matching technique, which allows to obtain the local density of states at the junction [6]. The Hamiltonian of the nanotube superlattice is directly diagonalized, yielding the band structure and wavefunctions of the infinite system [10].

## 4. Results 

### 4.1. Localization at Octagonal Defects

We begin by considering a diagonal junction between the (8,0) and (14,0) nanotubes, as shown in Figure 1(a). As discussed in the previous section, when joining the (14,0) tube to a diagonally cut (8,0) tube, an 8R octagonal defect appears at the back of the knee. At the kneecap we may have either (i) an 8N octagon or (ii) a pair of pentagons (2 × 5). This is visualized in the upper inset of Figure 1(a) and in Figure 1(b). In the latter case (ii) the pentagons mix the graphene sublattices, which causes the breaking of electron-hole symmetry.

Here we consider the case (i), that is, with two octagons, 8R and 8N, present at each junction. Since there are no pentagons, the system can be still considered as a bipartite lattice.

**Figure 2:**
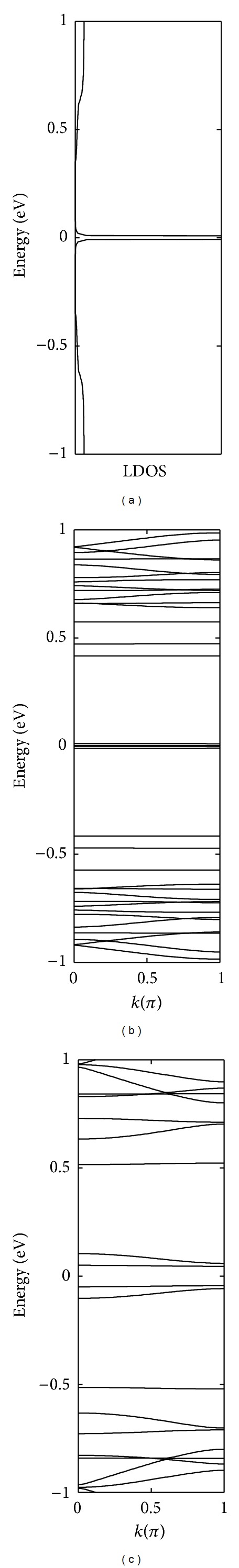
(a) LDOS at the wedge for the single junction (8,0)/(14,0). (b) Band structure of the 12(8,0)/12(14,0) SL. (c) Band structure of the 5(8,0)/5(14,0) SL.

Figure 2(a) shows the LDOS of an (8,0)/(14,0) single junction. A strong peak appears at *E* = 0 in the gap. In order to elucidate its origin, we perform calculations for two related superlattices *M*(8,0)/*M*(14,0) with *M* = 12 and *M* = 5 (*M* is the number of hexagons along the tube axis). The corresponding band structures are presented in Figures 2(b) and 2(c). Four bands, almost flat, appear near the Fermi level. They form two bonding and antibonding pairs because there are two junctions within a unit cell (recall Figure 1(c)). The wavefunctions of one pair are localized at the octagons 8R, whereas the wavefunctions of the other pair of bands are localized at the 8N octagons. The localization at the octagon 8N takes place at the sublattice to which the pair of nodes having only two neighbors belong. Consequently, localization at the octagon 8R occurs in the complementary sublattice. The wavefunctions centered at each octagon defect extend in a decaying way into the region of the complementary octagon but are always confined into their own sublattice. In the limit of *M* → *∞* we end up with the single junction case having a doubly degenerate *E* = 0 state, like the one shown in Figure 2(a).

Below we show that the appearance of states localized at the junctions is connected with the octagonal structure of the defects. To do this we divide the diagonal junction between the (8,0) and (14,0) tubes into three parts: (T1) a regular semi-infinite (8,0) tube, (W) the 62-atom wedge of the (8,0) tube, and (T2) a regular semi-infinite (14,0) tube, as shown in Figure 1.

According to the rules presented in [14], the semi-infinite (8,0) tube cut perpendicularly to its axis has three zero-energy edge states localized at the sublattice to which the zigzag-edge atoms belong. Similarly, the semi-infinite (14,0) tube has five *E* = 0 edge states localized at the same sublattice. On the other hand, the wedge portion has eight zero-energy states, since there is an imbalance of eight atoms belonging to different sublattices [22, 25]. All the wedge states are localized at the complementary sublattice with respect to the nanotube edges. Therefore, when all three parts, (T1), (W), and (T2), are connected by bonding the edge nodes of tubes with the edge nodes of the wedge, the zero-energy edge states should in principle mix, split, and merge into the bulk continuum. However, our calculations show that two zero-energy states are still present being localized at the octagonal defects 8R and 8N.

**Figure 3:**
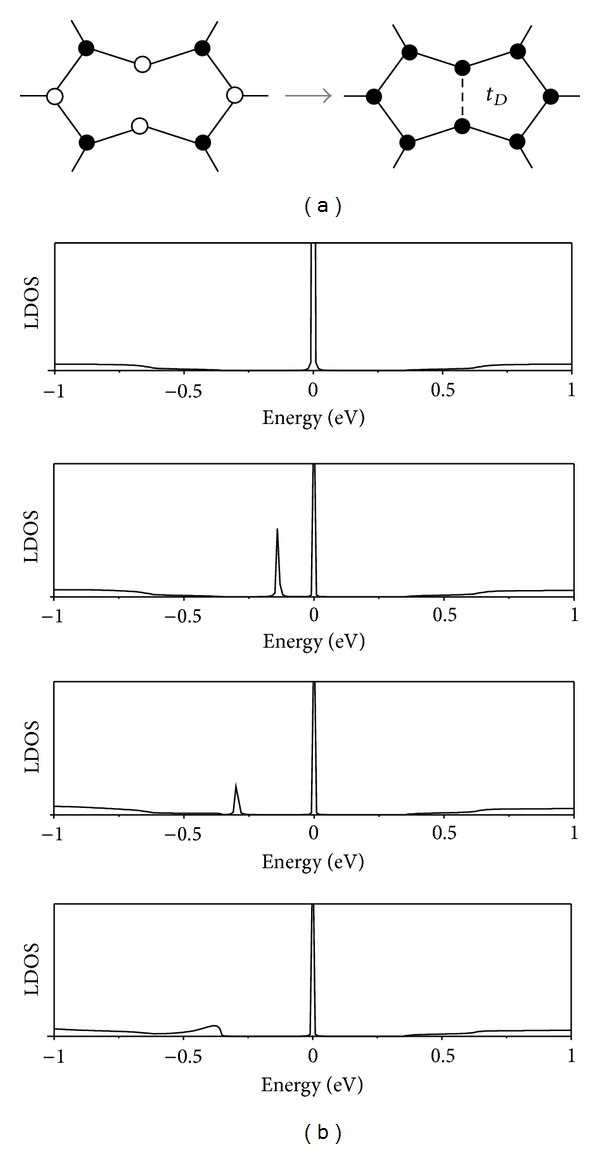
(a) A scheme showing the reconstruction of an 8N octagon into a pair of pentagons. (b) LDOS for the case of (8,0)/(14,0) junction with 8R octagon present and varying strengths of the bond between the two unsaturated nodes of the 8N octagon (hopping tD at this bond varies from top to bottom: 0, 0.2*t*, 0.5*t*, *t*).

The 8N octagon has two atoms with coordination number 2, so it can be expected that it may reconstruct into two pentagons. We thus connect the two unsaturated nodes of the 8N octagon, transforming it into a pair of pentagons (2 × 5) in the nanotube junctions, as shown in Figure 3(a).

Figure 3(b) shows the LDOS calculated at the wedge for increasing values of the hopping parameter *t*_*D*_ between these two nodes. The LDOS peak corresponding to the state localized at the octagon 8N moves from *E*_*F*_, lowers its energy, and finally merges into the bulk-band continuum. The peak corresponding to the localized state at the 8R octagon remains fixed at *E* = 0, because it is localized at the complementary sublattice. These results are in agreement with our previous calculations on nanotube SLs [26]. In these latter systems the Fermi level is below the bands localized at the junctions, so the octagon-localized states are unoccupied in this case.

### 4.2. Octagonal Defect and Its Void

**Figure 4:**
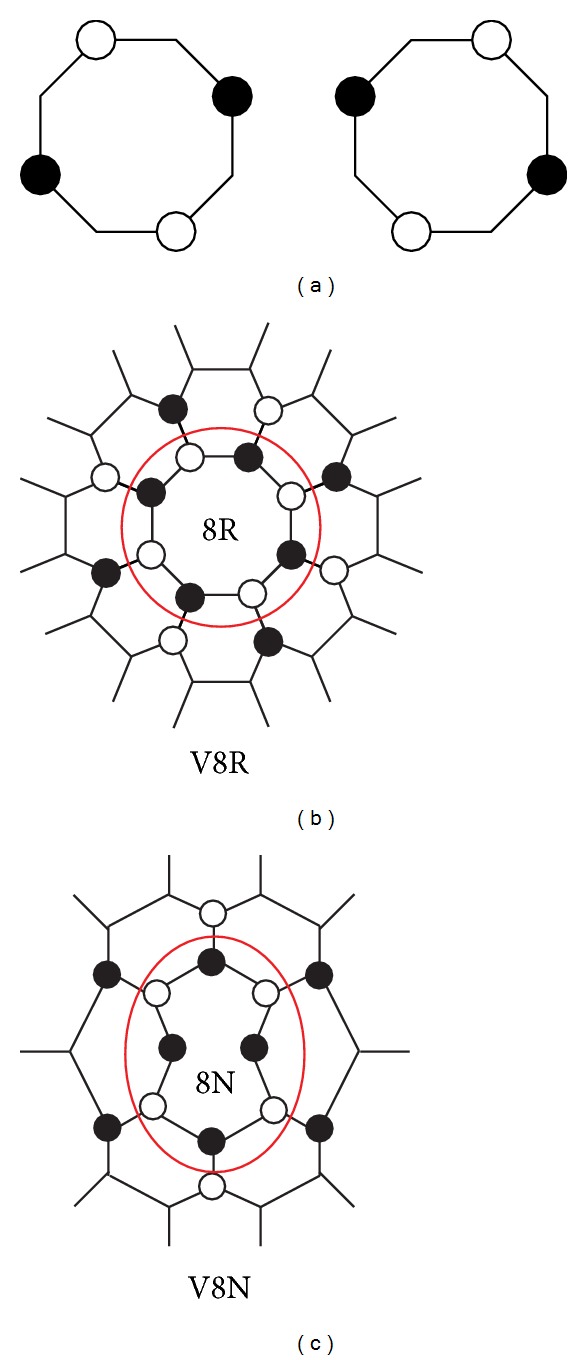
(a) Two possible representations of the zero-energy TB wavefunctions for the octagonal carbon ring. Filled and empty circles mean positive and negative values of the wavefunction; no circle means that the wavefunction is exactly zero. (b), (c) Schematic views showing how the 8R and 8N octagons are connected to the voids V8R and V8N, respectively. Filled and empty circles indicate the void-edge nodes corresponding to different sublattices.

**Figure 5:**
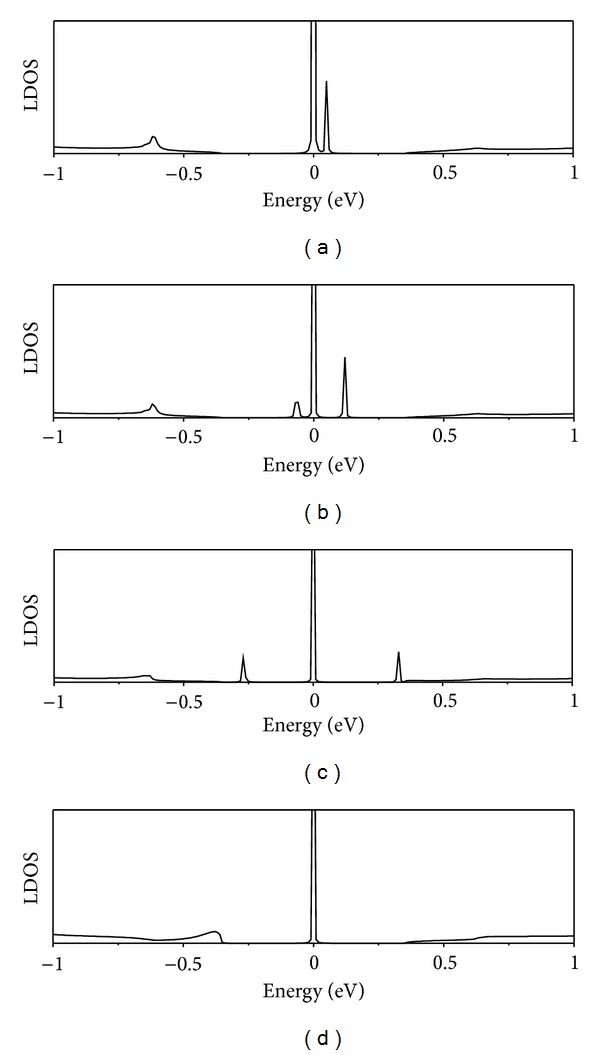
LDOS evaluated at the wedge region for the single (8,0)/(14,0) junction with a pair of pentagons (2 × 5) at the kneecap and varying strengths of the hopping $\tilde{t}$ parameter connecting the 8R octagon into the V8R void. The hopping parameter varies as 0, 0.1*t*, 0.4*t* and *t* from top to bottom.

Let us begin by remarking that an octagonal ring of carbon atoms has two zero-energy states. The two zero-energy TB functions, localized at different sublattices, are shown in Figure 4(a). We want to investigate how these two zero energy states evolve when they are connected to the rest of the knee-shaped structure. Let us consider the (8,0)/(14,0) junction with a pair of pentagons in the kneecap. If the 8R octagon at the back of the knee is disconnected from the lattice, setting to zero the hoppings $\tilde{t}$ between the octagon and the rest of the lattice, an 8-fold void (V8R) is left at the junction. The LDOS calculated at the wedge of this structure (i.e., the knee with a void plus the disconnected octagon) is shown at the topmost panel of Figure 5. The peak at *E* = 0 corresponds to a doubly degenerate zero-energy state of the detached octagon, while the peak at 0.05 eV represents the state localized at the edge of the V8R void. The wave function of this later peak is localized at one sublattice. Its energy is slightly displaced from *E*_*F*_ because of the presence of two pentagons that mix both sublattices and break electron-hole symmetry. This peak is roughly twice smaller than that at *E* = 0, because the central peak corresponds to a doubly degenerate energy level. When the octagon is connected to the void, one of its zero-energy states mixes with the void state, since they are localized at different sublattices. Those states split and merge into the bulk continuum, as shown in Figure 5. The peak corresponding to the other zero-energy state of the octagon is left unchanged. Its wavefunction spreads and decays out from the octagonal defect but remains in the same sublattice.

### 4.3. Effects of Electron Interaction

**Figure 6:**
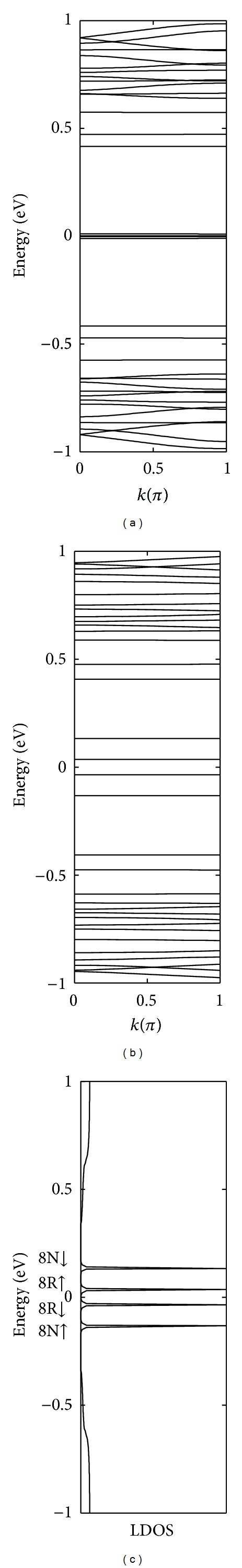
Comparison of band structures of 12(8,0)/12(14,0) SL calculated with the TB (a) and Hubbard (b) models. In the Hubbard model all bands are spin degenerate. (c) LDOS at a single junction, including the Coulomb repulsion (the peaks positions correspond to the flat bands presented at (b)). Arrows and letters R/N indicate spin polarization and localization at the 8R and 8N octagons, respectively.

As most of the studied structures present flat and degenerate bands at the Fermi level, we should consider the effect of electron-electron interactions on them. We employ the Hubbard model described in Section 3. In Figure 6 we show the bands of the 12(8,0)/12(14,0) SL calculated (a) with the TB model, (b) including the Hubbard term.  Figure 6(c) shows the LDOS corresponding to the (8,0)/(14,0) single junction including the Coulomb repulsion.

In both Figures 6(a) and 6(b) we see two pairs of bonding and antibonding bands near the Fermi level. They are more separated in energy when the Coulomb repulsion is taken into account. Bonding and antibonding bands are due to the presence of two junctions in the SL unit cell.

Note that when spin is included, the bands remain spin degenerate even in the Hubbard model, but the degeneracy is caused by the presence of two complementary junctions in the SL unit cell. Each band of a degenerate pair corresponds to a state located in the same defect but at a different junction, having opposite spins. Therefore, at each junction the states are not spin degenerate. This occurs also in the case of single junction, as shown in Figure 6(c). The four peaks at the LDOS of the single junction can be unequivocally related to the four bands of the SL. The spin splitting between the energy levels corresponding to the pair of spin-up and spin-down states localized at the 8R octagon is about 0.1 eV. For the octagon 8N this splitting is *≈*0.3 eV. The octagonal defects introduce local magnetic moments of different polarization at each octagon, thus rendering these systems antiferromagnetic. This finding can be compared to the antiferromagnetic ordering in zigzag graphene nanoribbons. In such systems the Coulomb interaction splits the four flat and edge-localized bands at *E* = 0 into two spin degenerate bands. Each of them corresponds to states located at different ribbon edges with opposite spin polarization. In contrast, in the nanotube junctions with two octagons, the zero energy states split into four different bands. This is because the octagons are not equal, so their states experience different Coulomb repulsions.

## 5. Summary and Conclusions

We have investigated the octagonal defects which appear at diagonal junctions between zigzag carbon nanotubes. We have chosen the (8,0) and (14,0) tubes, which is a particular case of the (2*n*, 0)/(4*n* − 2,0) junction. With such a choice both tubes are semiconductors, so the defect-localized states lie within the energy gap. Two different octagons, surrounded by hexagons, appear at the junction between the tubes. They are the source of state localization at the Fermi level. The 8R octagon has all atoms with coordination number 3, whereas the other one, namely, the 8N, has two unsaturated atoms, and it can be reconstructed into two pentagons. The junction with two octagonal defects presents two degenerate localized states at *E* = 0. These states are associated with the zero-energy states of the octagonal carbon ring. When the 8N octagon reconstruction takes place, the state localized at this defect splits and merges into the bulk continuum, while the other state, that is, the one localized at 8R octagon, is left at *E* = 0.

We have included also the electron-electron interaction effects to see how they influence these localized states. We find that the single junction with two octagonal defects presents spin-split states localized at different octagons, thus yielding an antiferromagnetic ground state of the junction system. The superlattice bands are spin degenerate, but with the two junctions having opposite spin configuration.
